# Validation of the Patient Health Questionnaire-9 for Major Depressive Disorder in the Occupational Health Setting

**DOI:** 10.1007/s10926-015-9607-0

**Published:** 2015-09-16

**Authors:** D. Volker, M. C. Zijlstra-Vlasveld, E. P. M. Brouwers, W. A. Homans, W. H. M. Emons, C. M. van der Feltz-Cornelis

**Affiliations:** Trimbos-Institute, Netherlands Institute of Mental Health and Addiction, PO Box 725, 3500 AS Utrecht, The Netherlands; Tranzo, Tilburg University, Tilburg, The Netherlands; Department of Methodology and Statistics, Tilburg University, Tilburg, The Netherlands; Top Clinical Centre for Body, Mind and Health, GGZ Breburg, Tilburg, The Netherlands; Bureau HHM, Enschede, The Netherlands

**Keywords:** PHQ-9, Major depressive disorder, Validation study, Occupational health setting

## Abstract

*Purpose* Because of the increased risk of long-term sickness leave for employees with a major depressive disorder (MDD), it is important for occupational health professionals to recognize depression in a timely manner. The Patient Health Questionnaire-9 (PHQ-9) has proven to be a reliable and valid instrument for screening MDD, but has not been validated in the occupational health setting. The aim of this study was to validate the PHQ-9 for MDD within a population of employees on sickness leave by using the MINI-International Neuropsychiatric Interview (MINI) as a gold standard. *Methods* Participants were recruited in collaboration with the occupational health service. The study sample consisted of 170 employees on sickness leave between 4 and 26 weeks who completed the PHQ-9 and were evaluated with the MINI by telephone. Sensitivity, specificity, positive and negative predictive value, efficiency and 95 % confidence intervals (95 % CIs) were calculated for all possible cut-off values. A receiver operator characteristics (ROC) analysis was computed for PHQ-9 score versus the MINI. *Results* The optimal cut-off value of the PHQ-9 was 10. This resulted in a sensitivity of 86.1 % [95 % CI (69.7–94.8)] and a specificity of 78.4 % [95 % CI (70.2–84.8)]. Based on the ROC analysis, the area under the curve for the PHQ-9 was 0.90 [SE = 0.02; 95 % CI (0.85–0.94)]. *Conclusion* The PHQ-9 shows good sensitivity and specificity as a screener for MDD within a population of employees on sickness leave.

## Introduction

Major depressive disorders (MDD) are highly associated with sickness leave, and lead to personal suffering and high societal costs [1, 2]. The yearly prevalence of MDD in the working population of the Netherlands is 4.8 % [3]. Moreover, employees with MDD are at risk for long-term sickness leave [4, 5]. Long-term sickness leave is responsible for enormous costs for patients, companies and society as a whole. The loss in productivity and the payments for disability benefits place a substantial burden on the economies of many developed countries [6].

Because of the increased risk of long-term sickness leave for employees with a MDD, it is important for occupational health professionals (e.g., occupational physicians) to be able to recognize depression and start or refer to treatment in a timely manner. Several studies have shown that it is difficult to recognize MDD, because patients do not always present themselves with mental health problems [7, 8]. As such, the availability of good screening instruments for depression among employees on sickness leave is important. For the occupational health (OH) setting, these instruments must be brief, easy to use and reliable and valid for the specific population.

The Patient Health Questionnaire (PHQ) is a short, self-report version of the Primary Care Evaluation of Mental Disorders (PRIME-MD) [9]. The PHQ-9, the depression subscale of the PHQ, is a reliable and valid instrument for screening MDD [10, 11]. Several studies have reported good psychometric qualities of the PHQ-9 in primary care settings as well as in the general population [11–14]. A meta-analysis showed that the optimal cut-off points for diagnosing depression with the PHQ-9 are between 8 and 11 [15].

The commonly used cut-off value for the PHQ-9 is 10 [10]. However, the optimal cut-off score may differ depending on the setting [15]. In a validation study of the PHQ-9 in primary care in the Netherlands, an optimal cut-off value of 6 was found [13]. Whereas, a validation study in the Netherlands among diabetes patients in specialized outpatients clinics found an optimal cut-off value of 12 [16]. It could be expected that in a population who is suffering from other physical conditions and symptoms a higher cut-off value of the PHQ-9 is needed because these symptoms could be recognized by the PHQ-9 as depressive symptoms, while in reality they are symptoms of other physical conditions.

### Rationale

To our knowledge, validation of the PHQ-9 in the OH setting has not yet been performed. For many people, working is an important aspect of daily life and absence of work is associated with social isolation or loss of daily routines, which are also symptoms of MDD [17, 18]. Furthermore, sick-listed employees often have other physical disorders or conditions with symptoms that can also occur as symptoms of MDD, such as pain and fatigue [19]. This may cause higher scores on the PHQ-9 in a population of sick-listed employees than in the general population. Therefore, it is possible that to correctly identify MDD within a population of sick-listed employees, a higher cut-off value is necessary. The aim of the current study is to validate the PHQ-9 for the OH setting by comparing the PHQ-9 with the Dutch version of the MINI-International Neuropsychiatric Interview (MINI) as the gold standard [20].

## Methods

### Design

This validation study was performed as part of a randomized controlled trial (RCT) evaluating cost-effectiveness of an e-health module embedded in collaborative occupational health care for common mental health disorders. The design of this RCT is described extensively elsewhere [21]. In February 2011, the medical ethics committee at the Institutions for Mental Health, Utrecht, the Netherlands, approved the study protocol. Data for this validation study were collected in the recruitment phase of the RCT.

### Setting

The study was conducted in an occupational health setting.

### Participants

Employees on sickness leave for any reason between 4 and 26 weeks received written information about the study from the occupational health service, together with an information leaflet from the Trimbos-institute, an informed consent form and a screener that contained the PHQ-9. They were asked to participate in the RCT, to sign the informed consent form and to return it together with the completed screener to the researchers if they agreed to participate in the study. For the RCT, employees with a positive score on the PHQ-9 were contacted by telephone for a diagnostic interview, the MINI [20]. For this validation study, during a period of 4 months in the recruitment phase of the RCT, employees with negative PHQ-9 score were also contacted for a diagnostic interview. Employees who could not be contacted for a diagnostic interview within 30 days were excluded from the validation study. The interviewers were blinded to the results of the screener.

### Measurement Instruments

#### Demographics

Age, gender and duration of sickness absence were assessed at the start of the study.

#### The PHQ-9

The PHQ-9 is the subscale for depression of the self-administered version of the PRIME-MD diagnostic instrument for common mental disorders [10]. The PHQ-9 contains nine questions corresponding to the nine DSM-IV symptoms for MDD during the past 14 days. The answer categories were based on a 4-point response scale, with the categories ‘not at all’ (0), ‘various days’ (1), ‘more than half of the days’ (2) and ‘nearly every day’ (3). As such, the summed PHQ-9 score could range from 0 to 27. A score of ≥5 is considered an indication of mild depression, a score of ≥10 moderate depression, a score of ≥15 moderately severe depression and a score of ≥20 is an indication of severe depression [10].

#### MINI-International Neuropsychiatric Interview

The MINI-International Neuropsychiatric Interview is a short structured diagnostic interview, developed jointly by psychiatrists and clinicians, for diagnosis of the most common DSM-IV and ICD-10 psychiatric disorders [20]. For the current study, a Dutch version of the interview was used [22]. The MINI includes 23 disorders, however for the current study, only the modules for depressive and anxiety disorders were used. All interviewers were trained in carrying out the interview and were able to consult a psychiatrist in case of diagnosis uncertainty.

### Statistical Analysis

First, the demographic characteristics and the mean PHQ-9 scores were compared between the group of employees who, according to the MINI, had MDD, and the employees who did not have MDD. Chi square tests and independent samples *t* tests were used to test for significant differences. It was expected that the mean PHQ-9 score was higher in the MINI MDD group than in the MINI non-MDD group. This supports the construct validity of the scale, using the “known groups” method [23]. Cohen’s *d* was calculated for reporting effect size [24].

The diagnostic validity of the PHQ-9 was analysed in terms of sensitivity, specificity, positive predictive value (PPV), negative predictive value (NPV) and efficiency for all possible cut-off values of the PHQ-9 ranging from 0 to 27. Youden’s *J* (=(sensitivity + specificity) − 1) was computed to find the optimal balance between sensitivity and specificity. The optimal cut-off value is the value for which *J* reaches its maximum.

Furthermore, to access precision, 95 % confidence intervals (95 % CI) were calculated for the sensitivity, specificity, PPV, NPV and efficiency for each cut-off value. The 95 % CIs were computed using the method suggested by Agresti and Coull because this method also produces accurate 95 % CIs for observed proportions close to 0 or 1 [25, 26]. For cut-off values at the extremes of the PHQ-9, the sample sizes were too small to calculate accurate 95 % CI for the NPVs and PPVs. Therefore, we only report the 95 % CI of the NPV and PPV if the sample sizes were ≥15 [25].

A receiver operating characteristic (ROC) analysis was performed, which calculated an area under the curve (AUC) for the PHQ-9. The AUC can be interpreted as the distinctive character of the tests, or the probability that a randomly chosen participant would be correctly distinguished based on their screening score [27].

The statistical analyses were performed in SPSS version 22.0 [28].

## Results

### Flowchart

**Fig. 1:**
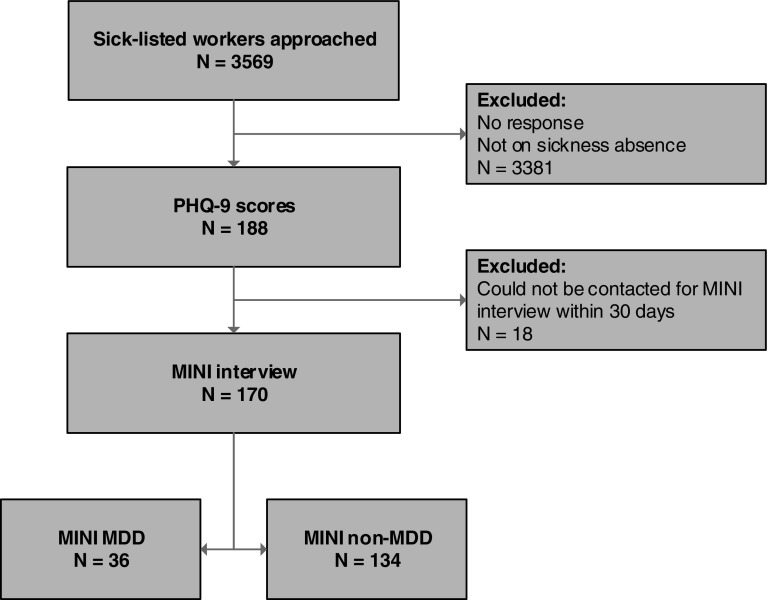
Flowchart of participants. *PHQ-9* The Patient Health Questionnaire-9, *MINI* MINI-International Neuropsychiatric Interview, *MDD* Major depressive disorder

In total, 3569 employees sick-listed due to any cause were approached to fill out the PHQ-9 questionnaire (and to participate in the RCT), of whom 188 employees returned the questionnaire. It is not known whether the 3381 non-responders had already fully returned to their work and therefore did not complete the PHQ-9 or that they did not respond due to any reason. Of the 188 eligible employees, 18 employees were unable to be reached for the MINI-interview within 30 days after they complete the PHQ-9. As a result, data from 170 employees were included in the analyses. From the total of 170 MINIs, 36 employees scored positively for MDD (prevalence = 21.2 %). Figure 1 shows the flowchart of the participants in this study.

### Demographic Characteristics

The mean age of participants in the final study sample (N = 170) was 45.4 years (SD = 10.9); age ranged from 21 to 66 years. Gender was divided equally between male and female participants (50.0 %). The average number of weeks of sickness leave when filling out the PHQ-9 was 10.8 (SD = 3.6). The average number of days between completion of the screener and administration of the MINI was 13.7 (SD = 7.2). None of these characteristics showed a significant difference between the MINI MDD and the MINI non-MDD group.

### Mean Scores PHQ-9

The mean score on the PHQ-9 for the entire group was 8.0 (SD = 7.1, range 0–27). The mean PHQ-9 score in the MINI MDD group was 16.3 (SD = 6.0, range 6–27) and the mean PHQ-9 score in the MINI non-MDD group was 5.8 (SD = 5.6, range 0–23). The difference between the means was significant (*p* < 0.01). This results in a Cohen’s *d* of 1.81, which indicates a large effect size [29].

### Classification Scores

Table 1 shows the sensitivity, specificity and corresponding 95 % CI for all possible cut-off values. Table 2 shows the predictive values for both positive and negative test results (PPV and NPV), efficiency and the corresponding 95 % CI for all the cut-off values of the PHQ-9.

**Table 1:** Sensitivity, specificity and 95 % CI of the PHQ-9

PHQ-9 score	Number of participants	Positive MINI	Sensitivity (%)	95 % CI	Specificity (%)	95 % CI
0	28	0	100	90.4–100	0	0.0–2.8
1	7	0	100	90.4–100	20.9	14.9–28.5
2	12	0	100	90.4–100	26.1	19.4–34.2
3	11	0	100	90.4–100	35.1	27.5–43.5
4	11	0	100	90.4–100	43.3	35.2–51.7
5	11	0	100	90.4–100	51.5	43.1–59.8
6	9	2	100	90.4–100	59.7	51.2–67.6
7	6	0	94.4	81.9–98.5	64.9	56.5–72.5
8	10	3	94.4	81.9–98.5	69.4	61.2–76.6
9	5	0	86.1	71.3–93.9	74.6	66.6–81.2
10	8	3	86.1	71.3–93.9	78.4	70.7–84.5
11	5	1	77.8	61.9–88.3	82.1	74.7–87.7
12	5	2	75.0	58.9–86.3	85.1	78.1–90.1
13	2	2	69.4	53.1–82.0	87.3	80.6–91.9
14	2	2	63.9	47.6–77.5	87.3	80.6–91.9
15	7	1	58.3	42.2–72.9	87.3	80.6–91.9
16	5	3	55.6	39.6–70.5	91.8	85.9–95.4
17	2	0	47.2	32.0–63.0	93.3	87.7–96.4
18	6	4	47.2	32.0–63.0	94.8	89.6–97.5
19	2	0	36.1	22.5–52.4	96.3	91.6–98.4
20	1	1	36.1	22.5–52.4	97.8	93.6–99.2
21	5	4	33.3	20.2–49.7	97.8	93.6-99.2
22	2	1	22.2	11.7–38.1	98.5	94.7–99.6
23	3	2	19.4	9.8–35.0	99.3	95.9–99.9
24	3	3	13.9	6.1–28.7	100	97.2–100
25	0	0	5.6	1.5–18.1	100	97.2–100
26	1	1	5.6	1.5–18.1	100	97.2–100
27	1	1	2.8	0.5–14.2	100	97.2–100

**Table 2:** PPV, NPV, efficiency and 95 % CI of the PHQ-9

PHQ-9 score	PPV (%)	95 % CI	NPV (%)	95 % CI	Efficiency	95 % CI
0	21.2	15.7–27.9	–	–	21.2	15.7–27.9
1	25.4	18.9–33.1	100	89.9–100	37.7	30.7–45.1
2	26.7	19.9–34.7	100	90.1–100	41.8	34.6–49.3
3	29.3	22.0–37.8	100	92.4–100	48.8	41.4–56.3
4	32.1	24.2–41.3	100	93.8–100	55.3	47.8–62.6
5	35.6	27.0–45.4	100	94.7–100	61.8	54.3–68.7
6	40.0	30.5–50.3	100	95.4–100	68.2	60.9–74.8
7	42.0	31.8–52.9	97.8	92.2–99.4	71.2	64.0–77.5
8	45.3	34.8–56.6	97.9	92.7–99.4	74.7	67.7–80.6
9	47.7	36.0–59.6	95.2	89.3–98.0	77.1	70.2–82.7
10	51.7	39.3–63.8	95.5	89.9–98.0	80.0	73.4–85.3
11	53.8	40.5–66.7	93.2	87.2–96.5	81.2	74.6–86.3
12	57.4	43.3–70.5	92.7	86.7–96.1	82.9	76.6–87.9
13	59.5	44.5–73.0	91.4	85.3–95.1	83.5	77.2–88.4
14	57.5	42.2–71.5	90.0	83.6–94.1	82.4	75.9–87.4
15	55.3	39.7–69.9	88.6	82.1–93.0	81.1	74.6–86.3
16	64.5	47.0–78.9	88.5	82.1–92.8	84.1	77.9–88.9
17	65.4	46.2–80.6	86.8	80.3–91.4	83.5	77.2–88.4
18	70.8	50.8–85.1	87.0	80.6–91.5	84.7	78.5–89.3
19	72.2	49.1–87.5	84.9	78.3–89.7	83.5	77.2–88.4
20	81.3	57.0–93.4	85.1	78.6–89.8	84.7	78.5–89.3
21	80.0	–	84.5	78.0–89.4	84.1	77.9–88.9
22	80.0	–	82.5	75.9–87.6	82.4	75.9–87.4
23	87.5	–	82.1	75.5–87.2	82.4	75.9–87.4
24	100	–	81.2	74.6–86.4	81.8	75.3–86.9
25	100	–	79.8	73.1–85.1	80.0	73.4–85.3
26	100	–	79.8	73.1–85.1	80.0	73.4–85.3
27	100	–	79.3	72.6–84.7	79.4	72.7–84.8

95 % CI for PPVs and NPVs based on <15 participants were not reported

Youden’s index *J* is highest at a cut-off value of 10. Table 1 shows that a cut-off value of 10 also results in the most optimal balance between sensitivity and specificity. This results in a sensitivity of 86.1 %, specificity of 78.4 %, PPV of 51.7 %, NPV of 95.5 % and an efficiency of 80.0 % (see Tables 1, 2).

### ROC Analysis

**Fig. 2:**
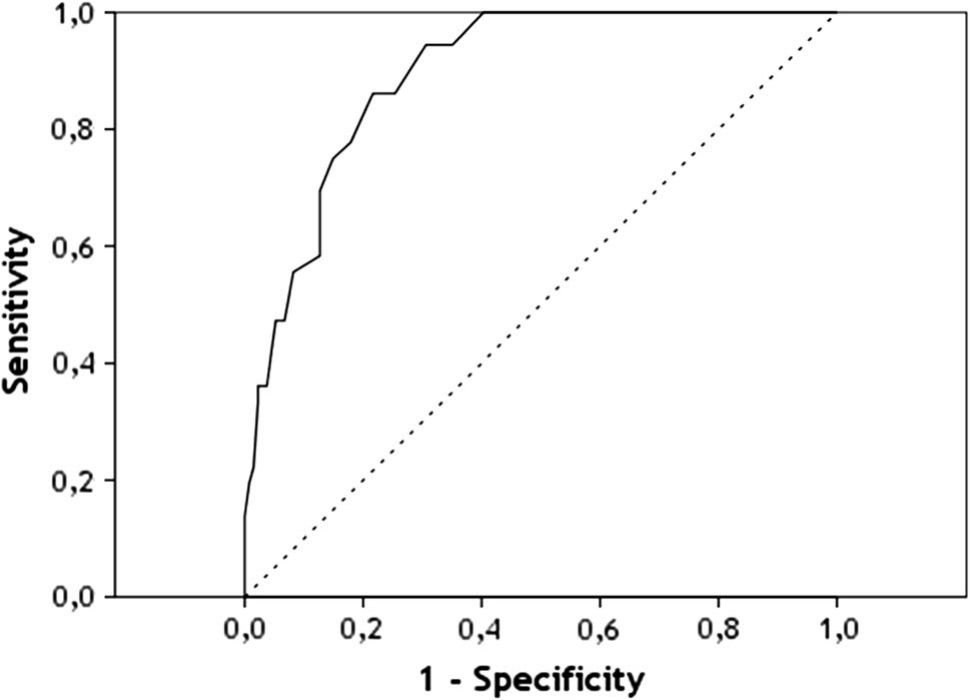
ROC-curve for the PHQ-9 versus MINI

The ROC curve is shown in Fig. 2. The calculated AUC for the PHQ-9 score versus the MINI was 0.90 [SE = 0.02; 95 % CI (0.85; 0.94)].

## Discussion

### Main Outcomes

In the current study, the concurrent validity of the PHQ-9 in screening MDD among sick-listed employees for any reason was evaluated. The mean scores on the PHQ-9 in the MINI MDD group versus the MINI non-MDD group were significantly different. This supports the construct validity of the PHQ-9. The PHQ-9 also showed good criterion validity characteristics; the optimal cut-off value was 10. At this value, the PHQ-9 has a sensitivity of 86.1 %, specificity of 78.4 %, PPV of 51.9 %, NPV of 95.5 % and efficiency of 80.0 %. This means that 86.1 % of sick-listed employees with MDD (according to the MINI), will be detected as such and 78.4 % of sick-listed employees without MDD will score negative on the PHQ-9. Furthermore, 51.9 % with a positive PHQ-9 score will be diagnosed with MDD by the MINI and 95.5 % with a negative PHQ-9 score will not be diagnosed with MDD by the MINI. The AUC refers to the distinctive character of the tests and is 0.90.

### Comparison with Other Studies

The optimal cut-off value for the PHQ-9 in this study was 10. This cut-off value is the same value that is typically used in primary care [10]. In a meta-analysis of validation studies of the PHQ-9, a pooled sensitivity of 85 % and a pooled specificity of 89 % was found for the cut-off value of 10 [15] this is comparable to the sensitivity and specificity that we found in the current study.

In the Netherlands, Zuithoff et al. [13] studied the validation of the PHQ-9 in a primary care setting. The results showed that the commonly used threshold of 10 had a sensitivity of 49 % and a specificity of 95 %. The optimal cut-off value was 6, which resulted in a sensitivity of 82 % and specificity of 82 % [13]. The fact that in the primary care setting in the Netherlands a lower cut-off value was found than in the OH setting could be due to the fact that sick-listed employees often have other physical disorders or conditions with symptoms that overlap with the symptoms of MDD. The PHQ-9 is also validated in the Netherlands in patients with diabetes in specialized outpatients clinics [16]. The optimal cut-off value in that setting was 12, which resulted in a sensitivity of 75.7 % and a specificity of 80.0 %. Thus, in that setting, a higher cut-off value was found than in the OH setting. It is hypothesized that this may be due to the fact that the patients from a specialized diabetes clinic have more severe pathology and more complications, which could be recognized by the PHQ-9 as depression symptoms, while instead being diabetes symptoms [16].

### Strengths and Limitations

A strength of the current study is that this was the first validation study of the PHQ-9 within a population of sick-listed employees. Another strength is that the interviewers were blinded to the results of the screener. The inclusion of the 95 % CIs using the method from Agresti and Coull is also a strength, as this indicates the precision of the estimated classification indices (e.g., sensitivity and specificity), which in turn informs researchers about the generalizability of the outcomes at the population level [25, 26].

A limitation of the current study is that because of the high rate of non-response and exclusion of participants that could not be reached within 30 days for the MINI-interview, selection bias might have occurred. Unfortunately, there were no demographic data for the non-responders; as a result a sensitivity analysis was impossible. Reasons for the high rate of non-response could be that this validation study was conducted alongside a randomized controlled trial and it is likely that employees who did not want to participate in the RCT did not respond to the screener. Furthermore, it is possible that a number of the employees did not respond to the screener because they were no longer on sick leave. Another limitation may be the amount of time between completion of the screener and the diagnostic interview. It is possible that the absence or presence of MDD at the time of completion of the screener did not match the results of the MINI-interview due to a change in symptoms in the time between the screener and the MINI-interview. However, the test–retest reliability of the PHQ-9 over a similar two week period, studied by Zuithoff et al. [13] was very good.

A final limitation is the lack of information about reason for sick leave, types of disabling conditions and comorbid physical symptoms of the sick-listed participants. However, Vlasveld et al. [5] showed that regardless of the reason for sick leave, depression is a predictor of a longer duration of absence from work. Therefore, it is important to detect MDD in this population of sick-listed employees regardless of their reason for absence.

### Practical and Research Implications

Our findings suggest that the PHQ-9 can be used as a screener for detecting MDD in the OH setting. The optimal cut-off value is determined by the decisions that are made based on the cut-off value and depend on the context in which the screening instrument is used. OPs often have to decide on the referral to treatment. It is important for them to save costs by avoiding unnecessary treatment and to refer to treatment correctly for the employees that need it. The test needs to detect the presence of the disorder in employees who actually suffer from the disorder, but it also needs to detect the absence of the disorder in a person who does not suffer from the disorder. It should be noted that with the cut-off value of 10, the PPV is 51.9 %, thus there is a substantial chance of false positives.

The PHQ-9 and MINI used in this study are both based on the DSM-IV; during the course of this study, the DSM-5 was published [30]. The criteria for MDD are minimally changed in the DSM-5, the most important change is that bereavement is no longer an exclusion criteria. The PHQ-9 scores are not affected by this change because the questionnaire does not include an item on bereavement. However, because the MINI does include a question about bereavement, the removal of bereavement as exclusion criterion for MDD might lead to a slightly better concurrent validity of the PHQ-9.

In the current study, the concurrent validity of the PHQ-9 in a population of sick-listed employees is studied. Further research could address other forms of validity testing and related aspects such as factor structure.

## Conclusions

Due to the increased risk of long-term sickness leave for employees with a MDD, it is important for occupational health professionals to recognize MDD and to start or refer to treatment in a timely fashion. This study showed that the PHQ-9 is a questionnaire with good sensitivity and specificity in the OH setting. Therefore, we recommend the use of the PHQ-9 as a screening instrument for MDD in sick-listed employees.

## References

[1] Laitinen-Krispijn S, Bijl RV (2000). Mental disorders and employee sickness absence: the NEMESIS study. Netherlands Mental Health Survey and Incidence Study. Soc Psychiatry Psychiatr Epidemiol.

[2] Henderson M, Glozier N, Holland EK (2005). Long term sickness absence. BMJ.

[3] Smit F, Cuijpers P, Oostenbrink J, Batelaan N, de Graaf R, Beekman A (2006). Costs of nine common mental disorders: implications for curative and preventive psychiatry. J Ment Health Policy Econ.

[4] Plaisier I, Beekman ATF, de Graaf R, Smit JH, van Dyck R, Penninx BWJH (2010). Work functioning in persons with depressive and anxiety disorders: the role of specific psychopathological characteristics. J Affect Disord.

[5] Vlasveld MC, van der Feltz-Cornelis CM, Bultmann U, Beekman AT, van Mechelen W, Hoedeman R (2011). Predicting return to work in workers with all-cause sickness absence greater than 4 weeks: a prospective cohort study. J Occup Rehabil.

[6] Henderson M, Harvey SB, Overland S, Mykletun A, Hotopf M (2011). Work and common psychiatric disorders. J R Soc Med.

[7] Lecrubier Y (2007). Widespread underrecognition and undertreatment of anxiety and mood disorders: results from 3 European studies. J Clin Psychiatry.

[8] Piek E, Nolen WA, Van der Meer K, Joling KJ, Kollen BJ, Penninx BWJH (2012). Determinants of (non-) recognition of depression by general practitioners: results of the Netherlands study of depression and anxiety. J Affect Disord.

[9] Spitzer RL, Williams JB, Kroenke K, Linzer M, deGruy FV, Hahn SR (1994). Utility of a new procedure for diagnosing mental disorders in primary care. The PRIME-MD 1000 study. J Am Med Assoc.

[10] Kroenke K, Spitzer RL, Williams JB (2001). The PHQ-9: validity of a brief depression severity measure. J Gen Intern Med.

[11] Gilbody S, Richards D, Brealey S, Hewitt C (2007). Screening for depression in medical settings with the Patient Health Questionnaire (PHQ): a diagnostic meta-analysis. J Gen Intern Med.

[12] Kroenke K, Spitzer RL, Williams JBW, Löwe B (2010). The Patient Health Questionnaire somatic, anxiety, and depressive symptom scales: a systematic review. Gen Hosp Psychiatry.

[13] Zuithoff NPA, Vergouwe Y, King M, Nazareth I, van Wezep MJ, Moons KGM (2010). The Patient Health Questionnaire-9 for detection of major depressive disorder in primary care: consequences of current thresholds in a crosssectional study. BMC Family Pract.

[14] Lowe B, Kroenke K, Herzog W, Grafe K (2004). Measuring depression outcome with a brief self-report instrument: sensitivity to change of the Patient Health Questionnaire (PHQ-9). J Affect Disord.

[15] Manea L, Gilbody S, McMillan D (2012). Optimal cut-off score for diagnosing depression with the Patient Health Questionnaire (PHQ-9): a meta-analysis. Can Med Assoc J.

[16] van Steenbergen-Weijenburg KM, de Vroege L, Ploeger RR, Brals JW, Vloedbeld MG, Veneman TF (2010). Validation of the PHQ-9 as a screening instrument for depression in diabetes patients in specialized outpatient clinics. BMC Health Serv Res.

[17] Bowling A (1995). What things are important in people’s lives? A survey of the public’s judgements to inform scales of health related quality of life. Soc Sci Med.

[18] Bilsker D, Wiseman S, Gilbert M (2006). Managing depression-related occupational disability: a pragmatic approach. Can J Psychiatry.

[19] Volker D, Zijlstra-Vlasveld MC, Brouwers EPM, van Lomwel AGC, van der Feltz-Cornelis CM (2015). Return-to-work self-efficacy and actual return to work among long-term sick-listed employees. J Occup Rehabil..

[20] Sheehan DV, Lecrubier Y, Sheehan KH, Amorim P, Janavs J, Weiller E (1998). The Mini-International Neuropsychiatric Interview (M.I.N.I.): the development and validation of a structured diagnostic psychiatric interview for DSM-IV and ICD-10. J Clin Psychiatry.

[21] Volker D, Vlasveld MC, Anema JR, Beekman ATF, Hakkaart-van Roijen L, Brouwers EPM (2013). Blended E-health module on return to work embedded in collaborative occupational health care for common mental disorders: design of a cluster randomized controlled trial. Neuropsychiatr Dis Treat.

[22] van Vliet IM, de Beurs E (2007). Het Mini Internationaal Neuropsychiatrisch Interview (MINI). Een kort gestructureerd diagnostisch psychiatrisch interview voor DSM-IV en ICD-10-stoornissen. Tijdschrift voor Psychiatrie.

[23] Devellis RF (2003). Scale development: theory and applications.

[24] Cohen J (1988). Statistical power analysis for the behavioral science.

[25] Agresti A, Coull BA (1998). Approximate is better than “Exact” for interval estimation of binomial proportions. Am Stat.

[26] de Vroege L, Emons WHM, Sijtsma K, Hoedeman R, van der Feltz-Cornelis CM (2014). Validation of the 4DSQ somatization subscale in the occupational health care setting as a screener. J Occup Rehabil.

[27] Hanley JA, McNeil BJ (1982). The meaning and use of the area under a receiver operating characteristic (ROC) curve. Radiology.

[28] IBM SPSS Statistics for Windows, Version 22.0. Armonk, NY: IBM Corp, 2013.

[29] Cohen J (1992). A power primer. Psychol Bull.

[30] American Psychiatric Association (2013). Diagnostic and statistical manual of mental disorders.

